# Population Genetics and New Insight into Range of CAG Repeats of Spinocerebellar Ataxia Type 3 in the Han Chinese Population

**DOI:** 10.1371/journal.pone.0134405

**Published:** 2015-08-12

**Authors:** Shi-Rui Gan, Wang Ni, Yi Dong, Ning Wang, Zhi-Ying Wu

**Affiliations:** 1 Department of Neurology and Research center of Neurology in Second Affiliated Hospital, and the Collaborative Innovation Center for Brain Science, Zhejiang University School of Medicine, Hangzhou, China; 2 Department of Neurology and Institute of Neurology, First Affiliated Hospital, Fujian Medical University, Fuzhou, China; 3 Department of Neurology and Institute of Neurology, Huashan Hospital, Institutes of Brain Science and State Key Laboratory of Medical Neurobiology, Shanghai Medical College, Fudan University, Shanghai, China; Emory University, UNITED STATES

## Abstract

Spinocerebellar ataxia type 3 (SCA3), also called Machado-Joseph disease (MJD), is one of the most common SCAs worldwide and caused by a CAG repeat expansion located in *ATXN3* gene. Based on the CAG repeat numbers, alleles of *ATXN3* can be divided into normal alleles (ANs), intermediate alleles (AIs) and expanded alleles (AEs). It was controversial whether the frequency of large normal alleles (large ANs) is related to the prevalence of SCA3 or not. And there were huge chaos in the comprehension of the specific numbers of the range of CAG repeats which is fundamental for genetic analysis of SCA3. To illustrate these issues, we made a novel CAG repeat ladder to detect CAG repeats of *ATXN3* in 1003 unrelated Chinese normal individuals and studied haplotypes defined by three single nucleotide polymorphisms (SNPs) closed to *ATXN3*. We found that the number of CAG repeats ranged from 13 to 49, among them, 14 was the most common number. Positive skew, the highest frequency of large ANs and 4 AIs which had never been reported before were found. Also, AEs and large ANs shared the same haplotypes defined by the SNPs. Based on these data and other related studies, we presumed that *de novo* mutations of *ATXN3* emerging from large ANs are at least one survival mechanisms of mutational *ATXN3* and we can redefine the range of CAG repeats as: ANs≤44, 45 ≤AIs ≤49 and AEs≥50.

## Introduction

Spinocerebellar ataxias (SCA) were a group of autosomal dominant ataxic disorders and spinocerebellar ataxia type 3 (SCA3) was regarded as one of the most common SCAs in the world [1, 2]. SCA3 was caused by a CAG repeat expansion located in exon10 of the *ATXN3* gene on chromosome 14q32.1 [3] and it was associated with a variety of clinical manifestations, including progressive ataxia, ophthalmoplegia, pyramidal signs, extrapyramidal signs and facial myokymia [4].

The relative frequency of SCA3 in SCAs was varied in different populations. Some studies have suggested that the frequency of large normal alleles (ANs) is related to the prevalence of SCA3 [5–11] and large ANs and expanded alleles (AEs) share the same haplotypes [8,10,12]. All these data supported the hypothesis that large ANs may constitute a reservoir from which AEs may emerge. However, a reverse result by detecting 1000 normal individuals in the Portuguese population with the highest prevalence of MJD globally was reported [13]. It seemed reasonable that there was no direct relationship between large ANs and the prevalence of SCA3 because of large samples [13].

For its complexity and heterogeneity in clinical manifestations, molecular genetic testing is the only way to make a definite diagnosis to SCA3. In addition, the definition of range of CAG repeats was a key point to interpreting the results of genetic testing as mentioned by some guidelines of genetic testing in SCAs [14–16]. The numbers of CAG repeats of *ATXN3* were first described as 13–36 in ANs and as 68–79 in AEs [3]. However, in recent publications [17–22] and even in guidelines of SCAs genetic testing [14,15], the range of CAG repeats had broadened to different numbers, which might lead to confusion in the molecular diagnosis of SCA3.

To illustrate this issue, we prepared a novel CAG repeat ladder, used it as a size marker to analyze the distribution of CAG repeats in 1003 unrelated Chinese normal individuals, and analyzed haplotypes defined by three SNPs closed to *ATXN3*. Based on the data of the current study and other related studies, we further redefined the range of CAG repeats in *ATXN3* to drive the genetic testing in Chinese patients with SCA3.

## Materials and Methods

### Subjects

One thousand and three unrelated normal individuals with no known history of hereditary disorders were recruited. Most of them (997/1003) were from eastern and southeastern China, including Fujian (595), Shanghai (330), Zhejiang (30), Jiangsu (28), Anhui (6), Jiangxi (4), Hunan (2) and Shandong (2). The remaining 6 were from Henan (3), Shanxi (1), Beijing (1) and Liaoning (1). Also, 30 unrelated SCA3 patients confirmed by molecular analysis of *ATXN3* were recruited for haplotypes studying. Written informed consent was obtained from each subject (if <18 years of age, written informed consent was obtained from their legal guardians), and the study was approved by the Ethics Committee of Huashan Hospital and First Affiliated Hospital. Genomic DNA was extracted from peripheral EDTA blood with a QIAamp DNA Blood Minikit (QIAGEN, Hilden, Germany).

### Identification of CAG repeats via DNA sequencing

The number of CAG repeats was identified via DNA sequencing in 200 out of the 1003 normal individuals. The CAG repeats expansion was amplified using MJD52/MJD25 primers and the procedure was as previously reported [23]. The amplified products were purified and subjected to direct sequencing using the procedure as previously reported [24].

### Preparation of a novel CAG repeat ladder

PCR products with different CAG repeats identified by DNA sequencing were cloned into a pMD18-T vector according to the manufacturer’s recommendations (TaKaRa, Chiba, Japan). The positive colonies were verified via PCR using MJD52/MJD25 primers [3] and the numbers of CAG repeats were further verified by DNA sequencing. The plasmids with different numbers of CAG repeats were mixed proportionally and used as a template to amplify the different CAG repeats expansion. This PCR product is a novel CAG repeat ladder containing a lot of bands and can be used as a DNA size marker to identify the number of CAG repeats in an 8% polyacrylamide gel (PAGE).

### Identification of CAG repeats via PAGE

The numbers of CAG repeats were identified via PAGE in the remaining 803 normal individuals. The prepared CAG repeat ladder was used as a DNA size marker, and the procedure of PAGE was used as previously reported [23]. When the transport ratio of a certain PCR product was different from all bands of the CAG repeat ladder, this PCR product was selected to clone into the pMD18-T vector and the procedure was repeated in order to broaden the range of the ladder. To measure the validity of the CAG repeat ladder, 30 samples were selected randomly from 803 normal individuals and their CAG repeats numbers were confirmed both PAGE and DNA sequencing.

### Analyses of SNPs genotype and haplotype

Three SNPs closely linked to the CAG repeats were studied. The genotype of A^669^TG/G^669^TG was detected by single-strand conformation polymorphism (SSCP) using MJD1VSR / MJD734R primers [25]. SSCP was performed in a polyacrylamide gel containing 5% glycerine. The gel was run at 27 V/cm at 20°C for 4.5 hours and silver stained to visualize the bands. The genotypes of the other two SNPs, C^987^GG/G^987^GG and TAA^1118^/TAC^1118^, were identified via allele-specific PCR using MJD-GGG or MJD-CGG and MJD-TAA or MJD-TAC, respectively, in combination with MJD-52 as a primer [12]. The PCR products were first separated by 2.5% agarose gel electrophoresis (AGE); if blurred, they were further separated by PAGE. The haplotype (partial haplotype) of these two SNPs was identified via AGE and PAGE, because the PCR products contained the CAG repeats expansion. However, the haplotype defined by all three SNPs (complete haplotype) could only be determined when the genotype of A^669^TG/G^669^TG was homozygous.

### Statistical analysis

All statistical analyses were performed using SPSS software version 11.0 (SPSS, Chicago, IL). The mean, median, variance, skewness and heterozygosity were determined for the distributions of ANs. In accordance with the previous report[5], the alleles carrying more than 27 CAG repeats (>27 repeats) were defined as large ANs. Chi-square tests were used to analyse the difference between present study and other studies both in the frequency of the large ANs and the relationship between CAG repeats number and the genotype and haplotype of SNPs. The results were considered statistically significant at p < 0.05.

## Results

### Analysis of CAG repeats ladder

**Fig 1:**
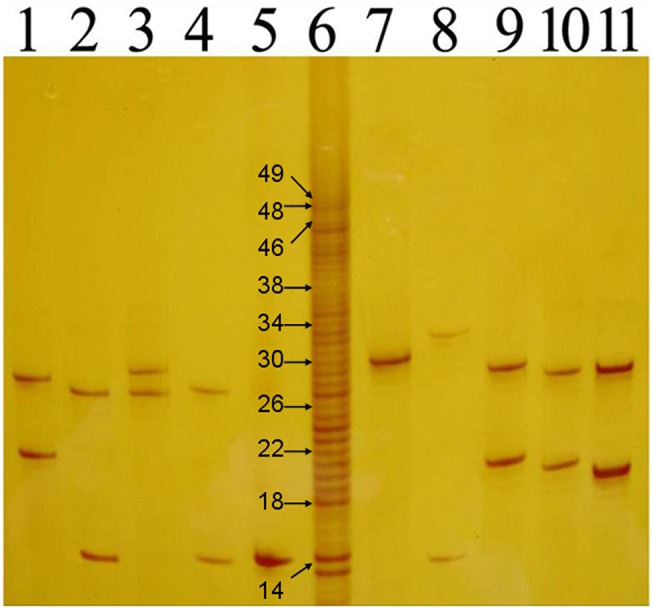
The polyacrylamide gel electrophoresis analysis of ANs and the CAG repeat ladder. Lanes 1–6 and 8–12 were ANs; lane 7 was the CAG repeat ladder. The CAG repeat numbers of the ladder’s bands were 13, 14, 18–43, 45, 46, 48 and 49. The bands contained 14, 18, 22, 26, 30, 34, 38, 46, 48 and 49 CAG repeats were marked by specific number.

As shown in Fig 1, the prepared CAG repeat ladder included 32 bands presenting different CAG repeat numbers. The different bands were distinct on PAGE. The CAG repeat numbers were 13, 14, 18–43, 45, 46, 48 and 49. The CAG repeats numbers of 30 selected samples were the same using both DNA sequencing and PAGE with DNA size marker of the CAG repeat ladder.

### Distribution of CAG repeats in 1003 normal individuals

**Fig 2:**
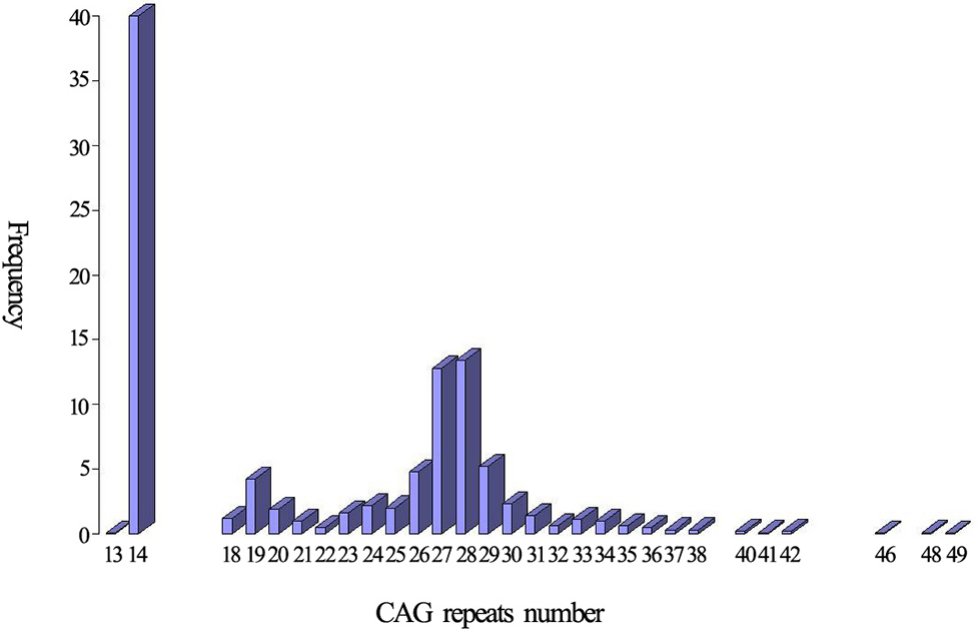
The distribution of the CAG repeats in 2006 wild-type chromosomes.

Twenty-nine alleles with the heterozygosity of 0.90 were identified in 1003 normal individuals. The distribution of the 29 alleles is shown in Fig 2. The number of CAG repeats ranged from 13 to 49. The three most frequent alleles had 14 (40.0%), 28 (13.4%) and 27 (12.8%) CAG repeats. The mean, median, variance and skewness were 21.70, 23.00, 50.42 and 0.22, respectively. Four individuals carried one of intermediate alleles (AIs) of which CAG repeats number were 46, 48, 48 and 49, respectively. Frequency of large ANs was 0.28(552/2006). The difference in the frequency of large ANs between the present study and other studies is shown in Table 1.

**Table 1:** The frequencies of large normal alleles of *ATXN3*.

Number of CAG repeats	Present study	Japanese			Indian			Czech			Combined Population		
	Frequency	Frequency	χ^2^	P	Frequency	χ^2^	P	Frequency	χ^2^	P	Frequency	χ^2^	P
>27	0.28	0.21	5.098	0.024	0.12	80.588	0.000	0.09	33.809	0.000	0.17	16.549	0.000
>28	0.14	0.11	2.150	0.143	0.08	23.948	0.000	0.04	15.293	0.000	0.11	4.133	0.042
>29	0.09	0.07	1.294	0.255	0.04	19.630	0.000	0.04	6.071	0.014	0.08	0.257	0.612
>30	0.07	0.05	0.950	0.330	0.03	15.404	0.000	0.03	4.275	0.039	0.07	0.023	0.880
>31	0.05	0.05	0.127	0.722	0.02	11.956	0.001	0.01	5.643	0.018	0.05	0.300	0.584

### Association of haplotypes with CAG repeats expansion

**Fig 3:**
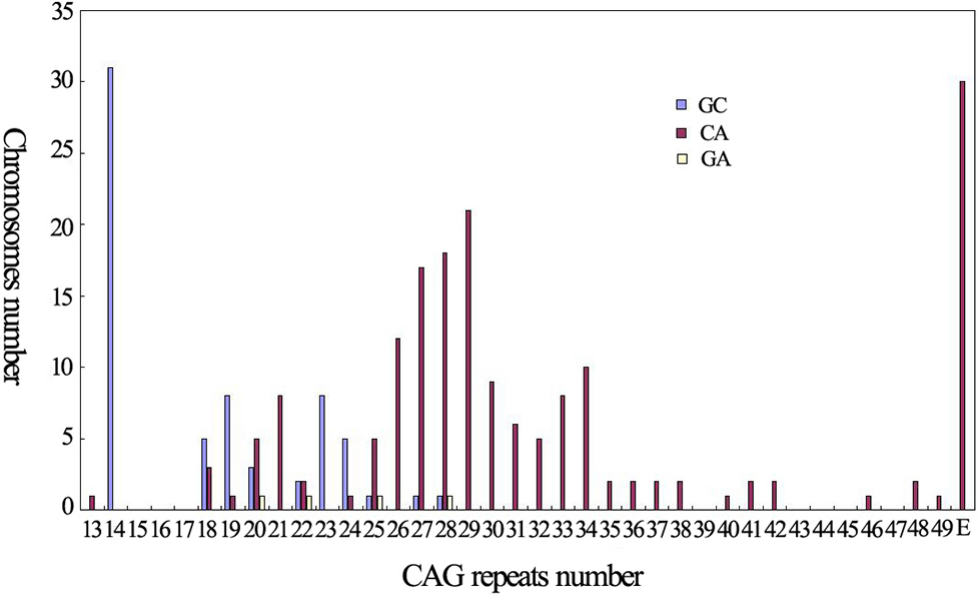
The distribution of the haplotypes defined by C^987^GG/G^987^GG and TAA^1118^/TAC^1118^ according to CAG repeat number.

A^669^TG/G^669^TG, C^987^GG/G^987^GG and TAA^1118^/TAC^1118^ were studied in 94 normal individuals and 30 SCA3 patients. Three partial haplotypes defined by C^987^GG/G^987^GG and TAA^1118^/TAC^1118^, CA (72.2%), GC (26.2%) and GA (1.6%) were found in all 124 subjects. The CA haplotype was significantly associated with large ANs compared to non-large ANs (**χ** ^**2**^ = 69.325, P<0.001). Actually, there were 94 CA haplotypes in all 96 partial haplotypes associated with large ANs, the other two haplotypes were GC and GA and both of which were associated with alleles contained 28 CAG repeats, and the haplotypes associated with AEs were exclusive CA (Fig 3 and Table 2).

**Table 2:** The frequencies of large normal alleles of *ATXN3*.

Alleles types	Partial haplotypes			Complete haplotypes			
	GA	GC	CA	GGC	AGA	AGC	ACA
**Non-large ANs**	3	64	55	2	2	11	32
**Large ANs**	1	1	94	0	1	1	74
**Expanded alleles**	0	0	30	0	0	0	15

Partial haplotypes were defined by C^987^GG/G^987^GG and TAA^1118^/TAC^1118^ and complete haplotypes were defined by A^669^TG/G^669^TG, C^987^GG/G^987^GG and TAA^1118^/TAC^1118^.

**Fig 4:**
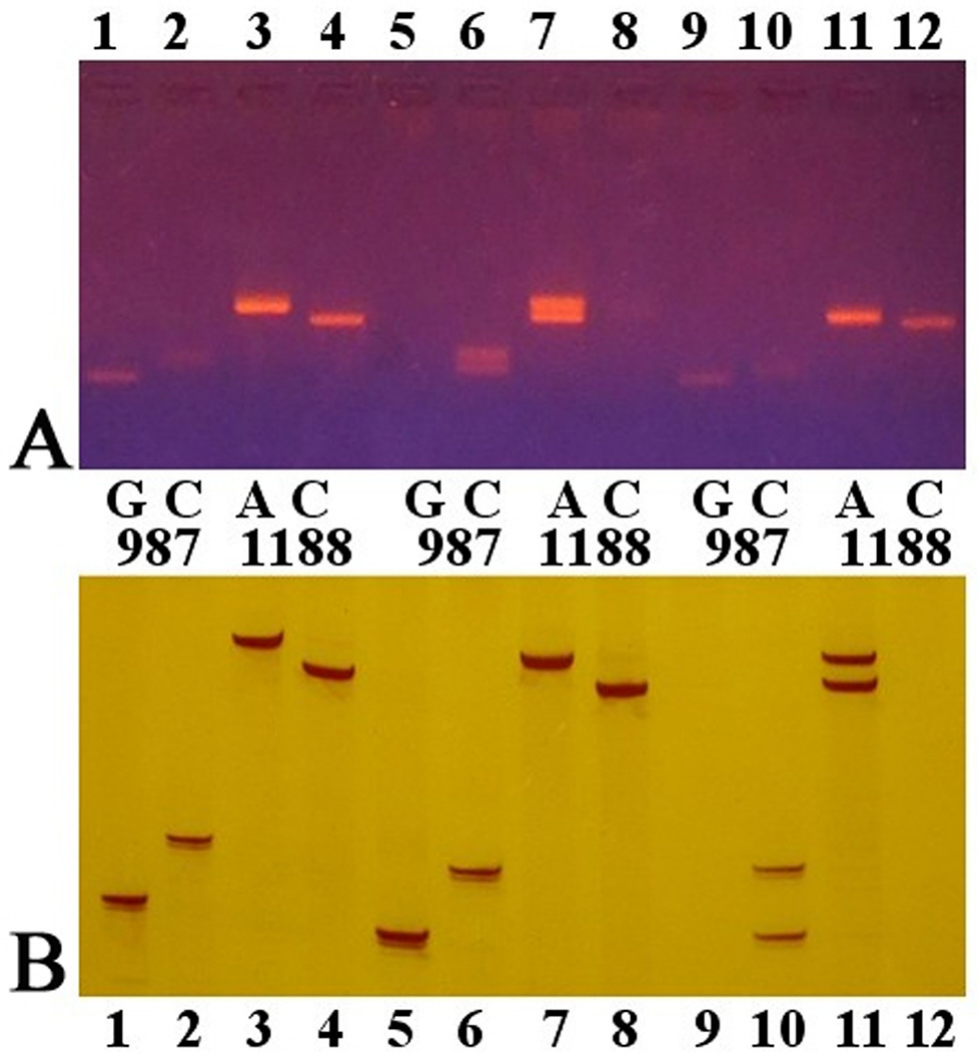
Detection of the haplotypes defined by C^987^GG/G^987^GG and TAA^1118^/TAC^1118^ via agarose gel electrophoresis (A) and polyacrylamide gel electrophoresis (B). Lanes 1–4, 5–8 and 9–12 in A and B were PCR products from 6 different individuals.

**Fig 5:**
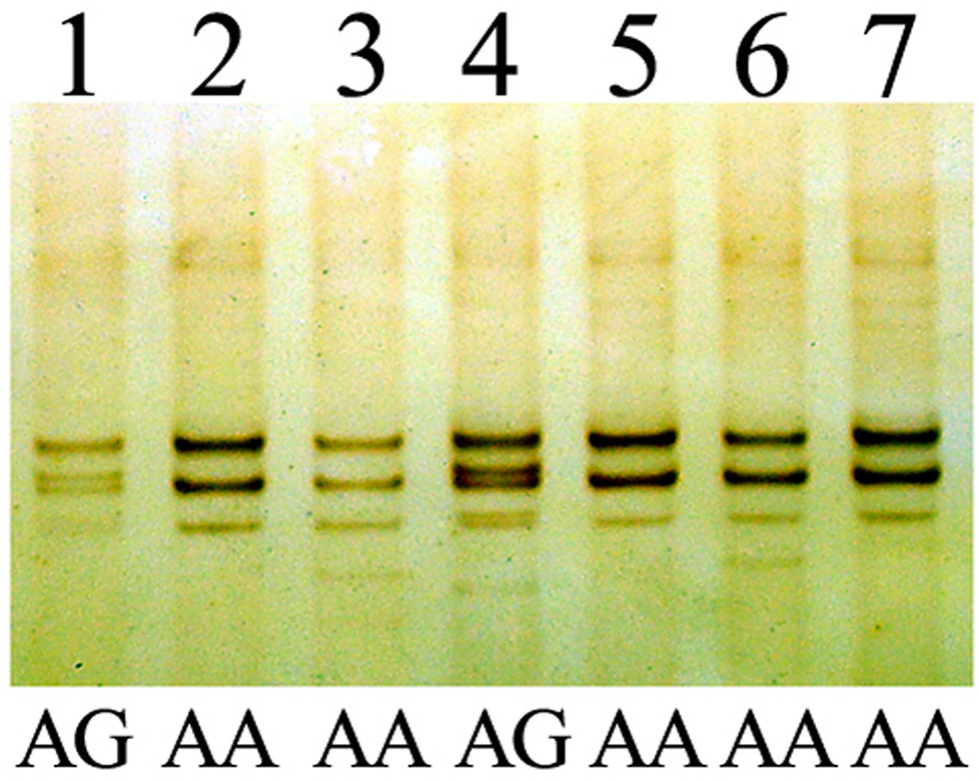
The single-strand conformation polymorphism analysis of PCR products in A^669^TG/G^669^TG. Lanes 1–7 were PCR products from different individuals.

Sixty-nine subjects were homozygotes at the SNP of A^669^TG/G^669^TG, 68 were homozygote of AA, and one was homozygote of GG. There were four complete haplotypes, ACA (87.7%), AGC (8.7%), AGA (2.2%) and GGC (1.4%), defined by all three SNPs in all 69 homozygotes. The ACA haplotype was significantly associated with large ANs compared to non-large ANs (χ^2^ = 20.908, P<0.001). There were 74 ACA haplotypes in all 76 complete haplotypes associated with large ANs, the other two haplotypes were AGC and AGA, both of which were associated with alleles contained 28 CAG repeats, and the haplotypes associated with AEs were exclusive ACA (Table 2). The agarose gel and polyacrylamide gel electrophoresis analysis of PCR products of C^987^GG/G^987^GG and TAA^1118^/TAC^1118^ are shown in Fig 4, respectively. The SSCP analysis of PCR products of A^669^TG/G^669^TG is shown in Fig 5.

## Discussion

It is well known that DNA sequencing, capillary electrophoresis (CE) and PAGE are the most common methods used to detect the CAG repeat number. DNA sequencing includes direct sequencing and sequencing after DNA recombination. Direct sequencing could detect the CAG repeat number accurately, but there are some difficulties caused by heterozygosity and somatic mosaicism that affect performance [26]. These difficulties could be avoided by DNA recombination, but the CAG repeat number may be altered in the procedure of DNA recombination [27, 28]. The benefit to using CE to detect CAG repeats is that it is highly efficient, and the drawback is that the detection cannot reflect the precise CAG repeat number [29]. Detecting CAG repeats via PAGE needs different kinds of DNA markers as size markers. The M13 DNA sequencing ladder is most widely used because of its precision, however it requires the rigorous conditions of electrophoresis and autoradiography to visualize the bands. To avoid these challenges, we prepared a novel and precise CAG repeat ladder by mixing the PCR products with different CAG repeat numbers. The disparity in size of each two consecutive bands was 3bp, therefore, the conditions of electrophoresis were not so rigorous compared to the M13 ladder. The different bands can be distinguished clearly by an 8% polyacrylamide gel and visualized by silver staining rather than autoradiography. And since the CAG repeats numbers were the same using both DNA sequencing and PAGE with the CAG repeat ladder, the accuracy of the CAG repeat ladder could be assured. Thus this novel ladder is an accurate and easily used size marker to detect the number of CAG repeats.

In the present study, we analyzed the characteristics of CAG repeats of *ATXN3* in 1003 normal individuals and found that the CAG repeat number ranged from 13 to 49 and 14 was the most common one. The frequency of large ANs (>27 repeats) was 0.28, which was the highest in all related reports as far as we known and significant higher than the frequency in populations of Japanese [5], Indian [8], Czechs [11] and combined population of Acadian, Black, Caucasian, Inuit and Thai [30]. In our previous study [23], the relative frequency of SCA3 in Chinese mainland population was also significant higher than that in these populations. Therefore, we presume that the frequency of large ANs is related to the prevalence of SCA3. In addition, the haplotypes of CA and ACA were both significant higher in large ANs than in non-large ANs, and the haplotypes of all AEs of SCA3 patients at present study were all CA or ACA. Base on these data, we suggest that large ANs may constitute a reservoir from which the AEs may be emerged in the Chinese population. Studies involved in SCA3 in different populations including Chinese Taiwan, Japanese, Australian, Indian, Czechs and French [5–12] or other diseases caused by dynamic mutation [31,32] also supported this hypothesis, however, the hypothesis did not sustain by another two studies involved in Portuguese in which the prevalence of SCA3 was the highest globally [13,25]. Therefore, it seems that the relationship between large ANs and AEs differ in different populations.

Intriguingly, we have such different results from that of Lima et al [13], though both involving in CAG distribution of large samples of normal individuals. We showed that large ANs and AEs were closely related, the distribution skewness which could reflect mutational bias [5,31] was positive and there were four AIs. However, Lima et al [13] found that large ANs were not related to the prevalence of SCA3, the skewness was negative and there were no AIs. In regard to the survival mechanism of mutational *ATXN3*, Lima et al [13] suggested that though larger AEs would be selected against because of early onset and severe symptoms, smaller AEs would be survival to maintain AEs in the populations since age of onset was after their reproductive period. However, this hypothesis seemed not to consider that even the smaller AEs would not survival since allele size of smaller AEs may increase successively over the generation sand lead to early onset [33]. The hypothesis also could not explain where AEs of sporadic SCA3 patients originate in different populations [34–37]. Therefore, we suggest that *de novo* mutations of *ATXN3* emerged from large ANs is at least one of survival mechanisms. Mittal et al [10] reported one AE with 45 CAG repeats may arise through gene conversion with one large AN and one smaller AN. Though *de novo* mutations of *ATXN3* that were direct evidence of our hypothesis yet reported, study of Mittal et al [10] supported our hypothesis indirectly. It is believable that *de novo* mutations of *ATXN3* exist since they have been found in some other dynamic mutation diseases such as Huntington’s disease (HD; MIM*#* 143100) [38], SCA2 (MIM*#* 183090) [39], SCA7 (MIM*#* 164500) [40] and SCA17 (MIM*#* 607136) [41]. The study should move on to find *de novo* mutations of *ATXN3* in next generations of carriers of large ANs and AIs.

The definition of range of CAG repeats is fundamental to genetic diagnosis of SCA3. The alleles of *ATXN3* can be divided into ANs that have never been associated with SCA3, AIs that are partially associated with SCA3 and AEs that are always associated with SCA3. When *ATXN3* was first cloned in 1994, the range of ANs and AEs were 13–36 and 68–79, respectively, and AIs were not found [3]. Since Tuite et al found one AE with 61 CAG repeats in 1995 [42] and Hsieh et al found one AN with 44 CAG repeats in 1997 [43], the range of ANs and AEs became ≤44 and ≥61, respectively, which was widely cited [26, 44]. Afterwards, many studies found AEs with CAG repeat numbers smaller than 61 [45–50], and 45 CAG repeats was the minimum for AE [50]. Also, Maciel et al reported one “AN” with 51 CAG repeats [26]. So, the range of CAG repeats was divided into ANs≤44, 45 ≤AIs ≤51 and AEs≥52, which was reported by Paulson [51]. However, this range has not been cited by the recent studies [14,15,17–22], except for our previous study [23]. Also, the range cited by the recent studies was different from one another. Therefore, there is a lot of confusion surrounding the range of CAG repeats.

In the current study, we found 4 individuals carrying 46, 48, 48 and 49 CAG repeats, respectively. Since none of them or their family members present cerebellar ataxia or other symptoms related to SCA3, the odds that these 4 individuals to be presymptomatic SCA3 patients is very rare. However, we will follow-up them to exclude presymptomatic states. Therefore, we suggest that the range of CAG repeats in *ATXN3* should be: ANs≤44, 45 ≤AIs ≤49 and AEs≥50. Intriguingly, there was a definition of mutable normal alleles (mutable ANs) except for ANs, AIs and AEs in *HTT* gene responsible for HD (MIM*#* 143100) [52]. Mutable ANs have not been associated with HD, but could increase to AIs or AEs to cause HD in the next generation. However, there is no definition of mutable ANs in *ATXN3* because no *de novo* mutation has been reported.

In summary, using the novel CAG repeat ladder, we detected CAG repeats of *ATXN3* in large Chinese population and found 4 AIs that had never been reported and the highest frequency (0.28) of large ANs. We presumed that *de novo* mutations of *ATXN3* is at least one survival mechanisms of mutational *ATXN3* since large ANs were so closed linked to AEs in Chinese population. And we redefine the range of CAG repeats in *ATXN3* as: ANs≤44, 45 ≤AIs ≤49 and AEs≥50.

## References

[1] Sequeiros J, Coutinho P. Epidemiology and clinical aspects of Machado–Joseph disease. Adv Neurol. 1993;61: 139–153. 8421964

[2] Schöls L, Bauer P, Schmidt T, Schulte T, Riess O. Autosomal dominant cerebellar ataxias: clinical features, genetics, and pathogenesis. Lancet Neurol. 2004;3:291–304. 15099544 10.1016/S1474-4422(04)00737-9

[3] Kawaguchi Y, Okamoto T, Taniwaki M, Aizawa M, Inoue M, Katayama S, et al CAG repeat expansions in a novel gene for Machado-Joseeph disease at chromosome 14q32.1. Nat Genet. 1994;8:221–228. 7874163 10.1038/ng1194-221

[4] Takiyama Y, Oyanagi S, Kawashima S, Sakamoto H, Saito K, Yoshida M, et al A clinical and pathologic study of a large Japanese family with Machado-Joseph disease tightly linked to the DNA markers on chromosome 14q. Neurology. 1994;44: 1302–1308. 8035935 10.1212/wnl.44.7.1302

[5] Takano H, Cancel G, Ikeuchi T, Lorenzetti D, Mawad R, Stevanin G, et al Close associations between prevalences of dominantly inherited spinocerebellar ataxias with CAG-repeat expansions and frequencies of large normal CAG alleles in Japanese and Caucasian populations. Am J Hum Genet. 1998;63: 1060–1066. 9758625 10.1086/302067 PMC1377499

[6] Saleem Q, Choudhry S, Mukerji M, Bashyam L, Padma MV, Chakravarthy A, et al Molecular analysis of autosomal dominant hereditary ataxias in the Indian population: high frequency of SCA2 and evidence for a common founder mutation. Hum Genet. 2000;106:179–187. 10746559 10.1007/s004390051026

[7] Storey E, du Sart D, Shaw JH, Lorentzos P, Kelly L, McKinley Gardner RJ, et al Frequency of Spinocerebellar Ataxia Types 1, 2, 3, 6, and 7 in Australian Patients with Spinocerebellar Ataxia. Am J Med Genet. 2000;95: 351–357. 11186889 10.1002/1096-8628(20001211)95:4<351::aid-ajmg10>3.0.co;2-r

[8] Chattopadhyay B, Basu P, Gangopadhyay PK, Mukherjee SC, Sinha KK, Chakraborty A, et al Variation of CAG repeats and two intragenic polymorphisms at SCA3 locus among Machado-Joseph disease/SCA3 patients and diverse normal populations from eastern India. Acta. Neurol Scand. 2003;108: 407–414. 14616293 10.1034/j.1600-0404.2003.00167.x

[9] Wu YR, Lin HY, Chen CM, Gwinn-Hardy K, Ro LS, Wang YC, et al Genetic testing in spinocerebellar ataxia in Taiwan: expansions of trinucleotide repeats in SCA8 and SCA17 are associated with typical Parkinson's disease. Clin Genet. 2004;65: 209–214. 14756671 10.1111/j.0009-9163.2004.00213.x

[10] Mittal U, Srivastava AK, Jain S, Jain S, Mukerji M. Founder haplotype for Machado-Joseph disease in the Indian population: novel insights from history and polymorphism studies. Arch Neurol. 2005;62: 637–640. 15824265 10.1001/archneur.62.4.637

[11] Bauer PO, Zumrova A, Matoska V, Marikova T, Krilova S, Boday A, et al Absence of spinocerebellar ataxia type 3/Machado–Joseph disease within ataxic patients in the Czech population. Eur J Neurol. 2005;12: 851–857. 16241973 10.1111/j.1468-1331.2005.01090.x

[12] Stevanin G, Lebre AS, Mathieux C, Cancel G, Abbas N, Didierjean O, et al Linkage disequilibrium between the spinocerebellar ataxia 3/Machado-Joseph disease mutation and two intrageneic polymorphisms, one of which, X359Y, affects the stop codon. Am J Hum Genet. 1997;60: 1548–1552. 9199580 10.1016/S0002-9297(07)64251-7 PMC1716136

[13] Lima M, Costa MC, Montiel R, Ferro A, Santos C, Silva C, et al Population genetics of wild-type CAG repeats in the Machado-Joseph disease gene in Portugal. Hum Hered. 2005;60: 156–163. 16340213 10.1159/000090035

[14] Seneca S, Morris MA, Patton S, Elles R, Sequeiros J. Experience and outcome of 3 years of a European EQA scheme for genetic testing of the spinocerebellar ataxias. Eur J Hum Genet. 2008;16: 913–920. 10.1038/ejhg.2008.29 18301445

[15] Sequeiros J, Seneca S, Martindale J. Consensus and controversies in best practices for molecular genetic testing of spinocerebellar ataxias. Eur J Hum Genet. 2010;18: 1188–1195. 10.1038/ejhg.2010.10 20179748 PMC2987480

[16] Sequeiros J, Martindale J, Seneca S, Giunti P, Kämäräinen O, Volpini V, et al EMQN Best Practice Guidelines for molecular genetic testing of SCAs. Eur J Hum Genet. 2010;18: 1173–1176. 10.1038/ejhg.2010.8 20179742 PMC2987475

[17] Long Z, Chen Z, Wang C, Huang F, Peng H, Hou X, et al Two novel SNPs in ATXN3 3' UTR may decrease age at onset of SCA3/MJD in Chinese patients. PLoS One. 2015;10: e0117488 10.1371/journal.pone.0117488 25689313 PMC4331546

[18] Mendonça LS, Nóbrega C, Hirai H, Kaspar BK, Pereira de Almeida L. Transplantation of cerebellar neural stem cells improves motor coordination and neuropathology in Machado-Joseph disease mice. Brain. 2015; 138: 320–335. 10.1093/brain/awu352 25527827

[19] Raposo M, Vasconcelos J, Bettencourt C, Kay T, Coutinho P, Lima M. Nystagmus as an early ocular alteration in Machado-Joseph disease (MJD/SCA3). BMC Neurol. 2014;14: 17 10.1186/1471-2377-14-17 24450306 PMC3901765

[20] Costa Mdo C, Luna-Cancalon K, Fischer S, Ashraf NS, Ouyang M, Dharia RM, et al Toward RNAi therapy for the polyglutamine disease Machado-Joseph disease.Mol Ther. 2013;21: 1898–1908. 10.1038/mt.2013.144 23765441 PMC3808129

[21] Pacheco LS, da Silveira AF, Trott A, Houenou LJ, Algarve TD, Belló C, et al Association between Machado-Joseph disease and oxidative stress biomarkers. Mutat Res. 2013;757: 99–103. 10.1016/j.mrgentox.2013.06.023 23994570

[22] Evers MM, Tran HD, Zalachoras I, Pepers BA, Meijer OC, den Dunnen JT, et al Ataxin-3 protein modification as a treatment strategy for spinocerebellar ataxia type 3: removal of the CAG containing exon. Neurobiol Dis. 2013;58: 49–56. 10.1016/j.nbd.2013.04.019 23659897

[23] Gan SR, Shi SS, Wu JJ, Wang N, Zhao G.X, Weng ST, et al High frequency of Machado-Joseph disease identified in southeastern Chinese kindreds with spinocerebellar ataxia. BMC Med Genet. 2012;11: 47. 10.1186/1471-2350-11-47 PMC2861663 20334689

[24] Wu ZY, Zhao GX, Chen WJ, Wang N, Wan B, Lin MT, et al Mutation analysis of 218 Chinese patients with Wilson disease revealed no correlation between the canine copper toxicosis gene MURR1 and Wilson disease. J Mol Med. 2006;84: 438–442. 16649058 10.1007/s00109-005-0036-y

[25] Maciel P, Gaspar C, Guimarães L, Goto J, Lopes-Cendes I, Hayes S, et al Study of three intragenic polymorphisms in the Machado-Joseph disease gene (MJD1) in relation to genetic instability of the (CAG)n tract. Eur J Hum Genet. 1999;7: 147–156. 10196697 10.1038/sj.ejhg.5200264

[26] Maciel P, Costa MC, Ferro A, Rousseau M, Santos CS, Gaspar C, et al Improvement in the molecular diagnosis of Machado-Joseph disease. Arch Neurol. 2001;58: 1821–1827. 11708990 10.1001/archneur.58.11.1821

[27] Jakupciak JP, Wells RD. Genetic Instabilities of Triplet Repeat Sequences by Recombination. IUBMB Life. 2000;50: 355–359. 11327307 10.1080/713803749

[28] Zheng QJ, Gan SR, Wang N, Wu ZY. The reliability of cloning-sequencing to detect the number of trinucleotide repeats. Zhonghua Shen Jing Ke Za Zhi. 2010;43: 659–663. (in Chinese)

[29] Dorschner MO, Barden D, Stephens K. Diagnosis of Five Spinocerebellar Ataxia Disorders by Multiplex Amplification and Capillary Electrophoresis. J Mol Diagn. 2002;2: 108–113. 10.1016/S1525-1578(10)60689-7 PMC1906987 11986402

[30] Limprasert P, Nouri N, Heyman RA, Nopparatana C, Kamonsilp M, Deininger PL, et al Analysis of CAG repeat of the Machado-Joseph gene in human, chimpanzee and monkey populations: a variant nucleotide is associated with the number of CAG repeats. Hum Mol Genet. 1996;5: 207–213. 8824876 10.1093/hmg/5.2.207

[31] Rubinsztein DC, Amos W, Leggo J, Goodburn S, Ramesar RS, Old J, et al Mutational bias provides a model for the evolution of Huntington’s disease and predicts a general increase in disease prevalence. Nat Genet. 1994;7: 525–530. 7951324 10.1038/ng0894-525

[32] Tishkoff SA, Goldman A, Calafell F, Speed WC, Deinard AS, Bonne-Tamir B, et al A global haplotype analysis of the myotonic dystrophy locus: implications for the evolution of modern humans and for the origin of myotonic dystrophy mutations. Am J Hum Genet. 1998;62:1389–1402. 9585589 10.1086/301861 PMC1377140

[33] Takiyama Y, Igarashi S, Rogaeva EA, Endo K, Rogaev EI, Tanaka H, et al Evidence for inter-generational instability in the CAG repeat in the MJD1 gene and for conserved haplotypes at flanking markers amongst Japanese and Caucasian. Hum Mol Genet. 1995;4: 1137–1146. 8528200 10.1093/hmg/4.7.1137

[34] Moseley ML, Benzow KA, Schut LJ, Bird TD, Gomez CM, Barkhaus PE, et al Incidence of dominant spinocerebellar and Friedreich triplet repeats among 361 ataxia families. Neurology. 1998;51: 1666–1671. 9855520 10.1212/wnl.51.6.1666

[35] Maruyama H., Izumi Y., Morino H., Oda M., Toji H., Nakamura S. et al Difference in disease-free survival curve and regional distribution according to subtype of spinocerebellar ataxia: a study of 1,286 Japanese patients. Am. J. Med. Genet. 114, 578–583 (2002). 12116198 10.1002/ajmg.10514

[36] Matsumura R, Futamura N, Ando N, Ueno S. Frequency of spinocerebellar ataxia mutations in the Kinki district of Japan. Acta Neurol Scand 2003;107: 38–41. 12542511 10.1034/j.1600-0404.2003.01347.x

[37] Jiang H, Tang BS, Xu B, Zhao GH, Shen L, Tang JG, et al Frequency analysis of autosomal dominant spinocerebellar ataxias in mainland Chinese patients and clinical and molecular characterization of spinocerebellar ataxia type 6. Chin Med J (Engl) 2005;118: 837–843. 15989765

[38] Goldberg YP, Kremer B, Andrew SE, Theilmann J, Graham RK, Squitieri F, et al Molecular analysis of new mutations for Huntington’s disease: intermediate alleles and sex of origin effects. Nat Genet. 1993;5: 174–179. 8252043 10.1038/ng1093-174

[39] Futamura N, Matsumura R, Fujimoto Y, Horikawa H, Suzumura A, Takayanagi T. CAG repeat expansions in patients with sporadic cerebellar ataxia. Acta Neurol Scand. 1998;98: 55–59. 9696528 10.1111/j.1600-0404.1998.tb07378.x

[40] Stevanin G, Giunti P, David G, Belal S, Dürr A, Ruberg M, et al De novo expansion of intermediate alleles in spinocerebellar ataxia 7. Hum Mol Genet. 1998;7: 1809–1813. 9736784 10.1093/hmg/7.11.1809

[41] Koide R, Kobayashi S, Shimohata T, Ikeuchi T, Maruyama M, Saito M, et al A neurological disease caused by an expanded CAG trinucleotide repeat in the TATA-binding protein gene: a new polyglutamine disease? Hum Mol Genet. 1999;8: 2047–2053. 10484774 10.1093/hmg/8.11.2047

[42] Tuite PJ, Rogaeva EA, St George-Hyslop PH, Lang AE. dopa-responsive parkinsonism phenotype of machado-joseph disease:confirmation of 14q CAG expansion. Ann Neurol. 1995;38: 684–687. 7574470 10.1002/ana.410380422

[43] Hsieh M, Tsai HF, Lu TM, Yang CY, Wu HM, Li SY. Studies of the CAG repeat in the Machado-Joseph disease gene in Taiwan. Hum Genet. 1997;100: 155–162. 9254842 10.1007/s004390050483

[44] Bettencourt C, Santos C, Kay T, Vasconcelos J, Lima M. Analysis of segregation patterns in Machado-Joseph disease pedigrees. J Hum Genet. 2008;53: 920–923. 10.1007/s10038-008-0330-y 18688568

[45] Takiyama Y, Sakoe K, Nakano I, Nishizawa M. Machado-Joseph disease: cerebellar ataxia and autonomic dysfunction in a patient with the shortest known expanded allele (56 CAG repeat units) of the MJD1 gene. Neurology 1997;49: 604–606. 9270607 10.1212/wnl.49.2.604

[46] van Schaik IN, Jöbsis GJ, Vermeulen M, Keizers H, Bolhuis PA, de Visser M. Machado-Joseph disease presenting as severe asymmetric proximal neuropathy. J Neurol Neurosurg Psychiatry. 1997;63: 534–536. 9343141 10.1136/jnnp.63.4.534 PMC2169790

[47] Egan RA, Camicioli R, Popovich BW. A small 55-repeat MJD1 CAG allele in a patient with Machado-Joseph disease and abnormal eye movements. Eur Neurol. 2000;44: 189–190. 11053973 10.1159/000008218

[48] van Alfen N, Sinke RJ, Zwarts MJ, Gabreëls-Festen A, Praamstra P, Kremer BP, et al Intermediate CAG repeat lengths (53, 54) for MJD/SCA3 are associated with an abnormal phenotype. Ann Neurol. 2001;49: 805–807. 11409435 10.1002/ana.1089

[49] Gu W, Ma H, Wang K, Jin M, Zhou Y, Liu X, et al The shortest expanded allele of the MJD1 gene in a Chinese MJD kindred with autonomic dysfunction. Eur Neurol. 2004;52: 107–111. 15316156 10.1159/000080221

[50] Padiath QS, Srivastava AK, Roy S, Jain S, Brahmachari SK. Identification of a novel 45 repeat unstable allele associated with a disease phenotype at the MJD1/ SCA3 locus. Am J Med Genet B. 2005;133: 124–126. 10.1002/ajmg.b.30088 15457499

[51] Paulson H. Spinocerebellar Ataxia Type 3. NCBI Bookshelf-GeneReviews. Available:http://www.ncbi.nlm.nih.gov/bookshelf/br.fcgi?book=gene&part=sca3.

[52] ACMG/ASHG Statement. Laboratory guidelines for Huntington disease genetic testing. Am J Hum Genet. 1998;62: 1243–1247. 9545416 PMC1377103

